# Exploring maternal nutrition counseling provided by health professionals during antenatal care follow-up: a qualitative study in Addis Ababa, Ethiopia-2019

**DOI:** 10.1186/s40795-021-00427-1

**Published:** 2021-06-07

**Authors:** Matyas Atnafu Alehegn, Tsegaye Kebede Fanta, Agumas Fentahun Ayalew

**Affiliations:** 1 Marie Stops International Ethiopia, Addis Ababa, Ethiopia; 2 GAMBY Medical and Business College, Addis Ababa, Ethiopia; 3 Department of Epidemiology and Biostatistics, Health Sciences College, School of Public Health, Woldia University, Woldia, Ethiopia

**Keywords:** Pregnant women, Counseling, Maternal nutrition, Antenatal care

## Abstract

**Background:**

Nutritional awareness and practice of women during pregnancy could be determining their nutritional status, which significantly affects the outcome of pregnancy. Therefore this study aims **t**o explore the maternal nutrition counseling provided by health professionals for pregnant women, Barriers to maternal nutrition, and major interventions.

**Methods:**

A descriptive study design with a qualitative method by using ground theory tradition, based on constructivist research approach and Charmaz’s (2000) study design has been conducted from September-01/2019 _November-16/2019 among pregnant women who got ANC service in Addis Ababa, Ethiopia. A purposive sampling technique was used. Practical observations and in-depth interviews were conducted. The sample size adjustment has been carried out according to the information saturation obtained, and finally, 81 practical observations, In-depth interview with two center managers, nine health professionals and eleven term pregnant women has been conducted. An observational checklist and Semi-structured, open-ended questionnaires were used. Data, the environment, and methodological triangulation were carried out. A conceptual framework has been established based on the data collected about the whole process of maternal nutrition counseling during pregnancy. ATLAS TI software was utilized for information analysis.

**The results:**

Most participants responded that maternal nutrition counseling provided to pregnant mothers is not adequate and neglected by most stakeholders. From 81 practical observations, health professionals counseled to mothers were 10 what to feed, 4 what to limit to consume, and 5 were counseled about what to eat during pregnancy. Close to all the respondents agreed on the importance of providing nutrition counseled by the nutritionists. Most of the study participants emphasized a shortage of time as primary barriers. Institutional Barriers, Professional Barriers, Maternal Barriers, and Community Barriers were major barriers to nutrition counseling.

**Conclusions:**

Generally, maternal nutrition counseling provided to pregnant mothers was not adequate and neglected by most stakeholders. Shortage of time due to client flow, Institutional Barriers, Professional Barriers, Maternal Barriers, and Community Barriers were major categories of maternal nutritional counseling barriers. Information update and timely preparation were recommended to health professionals.

## Background

Whole human beings require a balanced measure of foods for the proper performance of the body system. However, some factors can change nutrition requirements from individual to individual. For example, pregnancy is such a critical phase in a woman’s lifetime when the mother needs optimal nutrients of superior qualities to hold the growing fetus [1, 2].

Additionally, optimal levels of micronutrients are highly essential for women’s health during the childbearing years which is dependent on a mother’s intake or consumption. Although poor dietary intake is a common cause of micro-nutrient deficiencies, the root cause is associated with a poor socioeconomic level, and decreased knowledge about appropriate feeding style [3, 4].

Having decreased knowledge about nutrition and a balanced diet can be a problem to a healthy diet and proper use of supplements, mainly folic acid and iron [5].

A low level of nutritional awareness and practice of women during pregnancy can determine the nutritional status of pregnant women which significantly affects the outcome of pregnancy. Pregnant women were seen to have a low level of nutrition knowledge and practice and subjected to low adherence to iron foliate supplementation, especially in low-income countries like Ethiopia [3].

Pregnant women are in no position to make health and diet-related informed decisions during their course of pregnancy, so pregnant women need to have proper nutrition education from their Antenatal care (ANC) provider which reinforces good dietary behavior and practice. In developing nations like Ethiopia ANC providers are in a prime position to provide healthy eating information to pregnant women. While dietitians cited as a major source of nutrition information in western countries, pregnant mothers and family members are mentioned for developing ones [3].

Many factors hinder adequate dietary intake in pregnant and lactating women. Maternal food intake is usually dependent on individual choice, cultural beliefs; food taboos, beliefs surrounding pregnancy physiology, food aversions, household food availability, and economic constraints [6, 7].

Maternal nutrition education and counseling (NEC) on diet and weight gain, as well as monitoring of progress in maternal nutrition, are areas of needed attention. With the exception of special populations, and specific diets, NEC is normally implemented to enhance maternal dietary activity [4, 8].

For many pregnant women, dietary intake of vegetables, meat, dairy products, and fruit are often insufficient to meet these needs [9].

Social and behavior change interventions to improve maternal nutrition including maternal diet counseling can also be integrated into community-level activities, such as supervision conducted by health extension workers, or participating male and female community leaders through each duty [10].

As indicated by the Sustainable development Goals (SDGs) which is the United Nations (UN) agenda by the year 2030, nutrition is placed at the heart of the SDGs indeed, nutrition is vital for achieving twelve out of seventeen SDGs). The remaining five SDGs support improvements in nutrition [11].

The Health Sector Transformation Plan of the Ethiopian government stated that the focus of the country is to improve the nutritional status of mothers and children. Improve the nutritional status of women (15–49 years) and adolescent girls (10–19 years) are one of the strategic objectives stated in NNP-II [12].

Although healthcare practitioners perceived nutrition education to be important, because of many barriers to providing education to clients, generally, women are not receiving adequate nutrition education during Ante-Natal Care follow-up [13].

Counseling given to pregnant women regards nutrition was insufficient in opportunity and range. The counseling was characterized by a shortage of time, inadequate space, rare counseling, and interrupted documentation. Furthermore, counseling was provided only once to the mothers throughout ANC follow-up, that is at their first visit only [14].

According to the study done in the Oromia region, Ethiopia, even though there is a significant relationship between nutrition information and nutrition practices of mothers during pregnancy, pregnant mothers’ access to nutritional information is relatively low [1].

In Addis Ababa, the proportion of mothers who have appropriate knowledge of maternal nutrition during pregnancy was found to be 53.9% after proper nutrition education has been provided to them [3].

Even if, maternal nutrition requires considerable attention during pregnancy; nutritional counseling provided by a health professional during antenatal care (ANC) is not satisfactory [14] [5]. Many studies conducted around the globe, particularly in Africa and Asia reported that; Counseling given to pregnant women regard nutrition was insufficient in opportunity and range. The counseling was characterized by a shortage of time, inadequate space, rare counseling, and interrupted documentation. Previously conducted studies focus mainly on the maternal nutritional status and barriers of maternal education during pregnancy; which lacks to indicate the whole process of nutrition counseling and education, including interventions for barriers [3, 5, 10–12] [3]., [5], [13], [14], [15].

Therefore The purpose of this study is to explore the maternal nutrition counseling process for pregnant mothers holistically from current status up-to possible solutions for barriers of maternal nutrition counseling during pregnancy by indicating barriers, time and places of counseling, appropriate information to be included as well as possible interventions to be taken for improved maternal nutrition counseling during ANC follow-up.

Additionally, the result of this research can be used as a guide for MCH specialty centers and other institutions which delivering maternity services, for health care delivery, and teaching institutions to plan, prepare, conduct nutritional training for health professionals, and to rearrange settings of maternal nutrition counseling and education programs**.**

## Methods

### Study area

The research was conducted on three maternal and child health (MCH) specialty centers located in Addis Ababa, the capital city of Ethiopia. Currently, the total population of this city is expected to be more than 6.5 million with a high annual growth rate of 3.8%. Per the population recorded in the last census; the city of Addis Ababa has a higher population of female residents than male residents. More than thirty MCH specialty centers are found in Addis Ababa. The three MCH specialty centers are representatives of government, private and Non-governmental organizations (NGOs). Gandhi Memorial Hospital from the government institutions, Brass MCH specialty centers from private sectors, and Marie Stopes International MCH specialty centers from NGOs were selected to assure environmental triangulation and transferability of the study taking into consideration that; the three MCH service delivery provision institutions are providing the service to all social classes of the Community of Addis Ababa.

### Study design and period

A descriptive study design with a qualitative method by using ground theory was implemented, based on the constructivist research approach and Charmaz’s (2000) study design has been conducted from September-01/2019 _November-16/2019.

### Sampling technique, and sampling procedure

A purposive sampling technique was used. Eighty-one practical observations content were conducted using an observational checklist. The in-depth interview has been conducted with two center managers, nine health professionals, and eleven term pregnant women. The sample size adjustment has been carried out according to the information saturation obtained.

### Inclusion and exclusion criteria

Center managers, health professionals working in the outpatient department for ANC follow-up, and term pregnant women were included. Short-term contract staff members and pregnant women who are sick during the research period were excluded.

### Data collection procedures and quality assurance

Practical observation and in-depth interviews have been used. All practical observation and in-depth interviews were conducted by the principal investigator. First, the practical observation method used by an observational checklist, and then Semi-structured, open-ended questionnaires were administered to center managers, health professionals, and term pregnant women. Practical observation conducted before the in-depth interview of all participants in each center to prevent the bias of respondents. The specific topic was hidden during practical observation and the time was arranged between 9:00 AM and 3:00 PM when there is high client flow. The observational checklist prepared for this purpose was carefully filled by the main investigator and all important events have been documented during observation.

During practical site observation, the most important ANC activities stated by WHO-2016 ANC guidelines were checked. The checklist had two main categories of activities; ANC services and nutrition-related issues. Both of these categories had their own two subsections. Each activity was filled through tick mark and had space for recording of any activities performed during observation.

The in-depth interview with term pregnant mothers, health professionals, and center managers were carried out in places where there is no sound disturbance to an audio recording. Center manager offices, duty rooms, OPD examination rooms, and meeting halls were used for face-to-face interviews of participants. The time of the interview has been arranged according to the convenient time of the study participants, but the most preferred time was late the afternoon after 4:00 PM for professionals and morning before 11:00 AM to pregnant mothers, from 30 to 60 min for each participant.

Interview questionnaires were somewhat modified for pregnant mothers and center managers. The interview was conducted until information saturation was proved by the investigator. The pre-test has been conducted at the Family Guidance Association of Ethiopia (FGAE) MCH specialty center, which is found in Kirkos Sub-city. Two practical observations and one in-depth interview were conducted to check the clarity and understandability of the tool by study participants and data collectors.

### Data management and data analysis

ATLAS TI software and Microsoft Excel were used whenever necessary, All observational checklists have been analyzed using Microsoft excels and summarized by tables and graphs. Interviews data were translated and transcribed verbatim concurrently. The recorded in-depth interviews were transcribed verbatim. Open-coding has been conducted. As soon as a line-by-line coding, axial coding was applied to distinguish the main and sub-categories of the data. Data, environmental and methodological triangulation have been carried out throughout the research process. Finally, this study is ended-up by developing a conceptual framework based on the tradition of grounded theory.

## Results

### Observation

Only very few activities performed by health professionals during practical observation and counseling sessions have been done related to maternal nutrition issues, More than 230 major ANC activities and counseling sessions should be conducted, whereas, only less than 40 activities and counseling sessions were done in relation to maternal nutrition from 81 practical observations. On the other hand, food, and nutrition issues were highly neglected by health professionals (Tables 1, 2, and 3).

**Table 1:** Activities performed by health professionals during practical site observation

S.NO.	Activities	Performance/81 observations	Percent of activity
1	Greetings by Clients	75	93
2	Greetings by Health Professionals	79	98
3	Vital signs Measured	81	100
4	Weight of mothers measured	81	100
5	Fetal Movement Checked	81	100
6	Ultrasound scanning done	74	91
7	Laboratory Tests requested	78	96
8	TT vaccination ordered (Checked)	40	49
9	Physical Exercise is recommended to prevent varicose vein and Pelvic pain	4	5
10	Non-Pharmacology options recommended to prevent varicose vein & edema	0	–
11	Treatment of Diseases given, if there is any	6	7
12	What to eat is counseled	10	12
13	What not to eat is counseled	4	5
14	What to limit to eat is counseled	5	6
15	Recommended physical exercise is informed	7	9
16	Increasing daily energy and protein intake is recommended	9	11
17	Daily oral Ferrous plus Folic Acid supplementation recommended	38	47
18	Mothers asked about Substance abuse	3	4
19	Treatment or counseling is given to relief Nausea	1	1
20	Treatment or counseling is given to relief Heartburn	0	–
21	Treatment or counseling is given to relief Constipation	0	–
22	Maternal and fetal weight calculated/estimated and diet informed	25	31
23	Departure Greeting	79	98
24	Do clients say Thank you	79	98

**Table 2:** Major non-nutrition related activities performed by health professionals

S.NO.	Major activities	Performance/81 observations	Percent of activities
1	Next appointment told	48	59
2	Danger signs told	40	49
3	Gestational age of the fetus told	37	46
4	Laboratory results told	32	40
5	Fetal condition told	31	38
6	Fetal position told	24	30
7	Options of modes of delivery counseled	19	23

**Table 3:** nutrition activities performed by health professionals

S.NO.	Nutrition activities	Performance/81 observations	Percent of activities
1	Benefit of Iron, Folic Acid and vitamin Supplementation Counseled	2	2
2	Counseling is given about weight management related to nutrition	1	1
3	Counseling is given how and when to eat	1	1
4	Counseling is given to continue Iron Supplementation	6	7
5	Counseling is given to take food items less in quantity and more frequently	1	1
6	Counseling is given to eat home-made food items	5	6
7	Counseling is given to eat only cooked and clean food items	1	1
8	Counseling is given to eat too much and appropriate food to increase maternal weight gain	1	1
9	Counseling is given to prepare food item in a clean manner	1	1
10	Counseling is given to take plenty of fluids and/or clean water	9	11
11	Counseling is given to take better quantity of food	1	1
12	Counseling when and how to take iron supplementation is given	4	5
13	GI disturbance related to Iron Supplementation counseled	2	2
14	Importance of Folic Acid counseled	1	1
15	Information about iron rich food items is provided	1	1
16	Counseling is given to Limit coffee and tea	1	1

For example, what to eat, what to limit to eat, and what not to eat during pregnancy were only counseled to 10, 4, and 5 mothers respectively among 81 practical observations. Nothing is done or said about how to relieve heartburn and constipation. Only one mother had been counseled on how to relieve nausea during the whole observation period of pregnant mothers’ ANC visit.

**Fig. 1:**
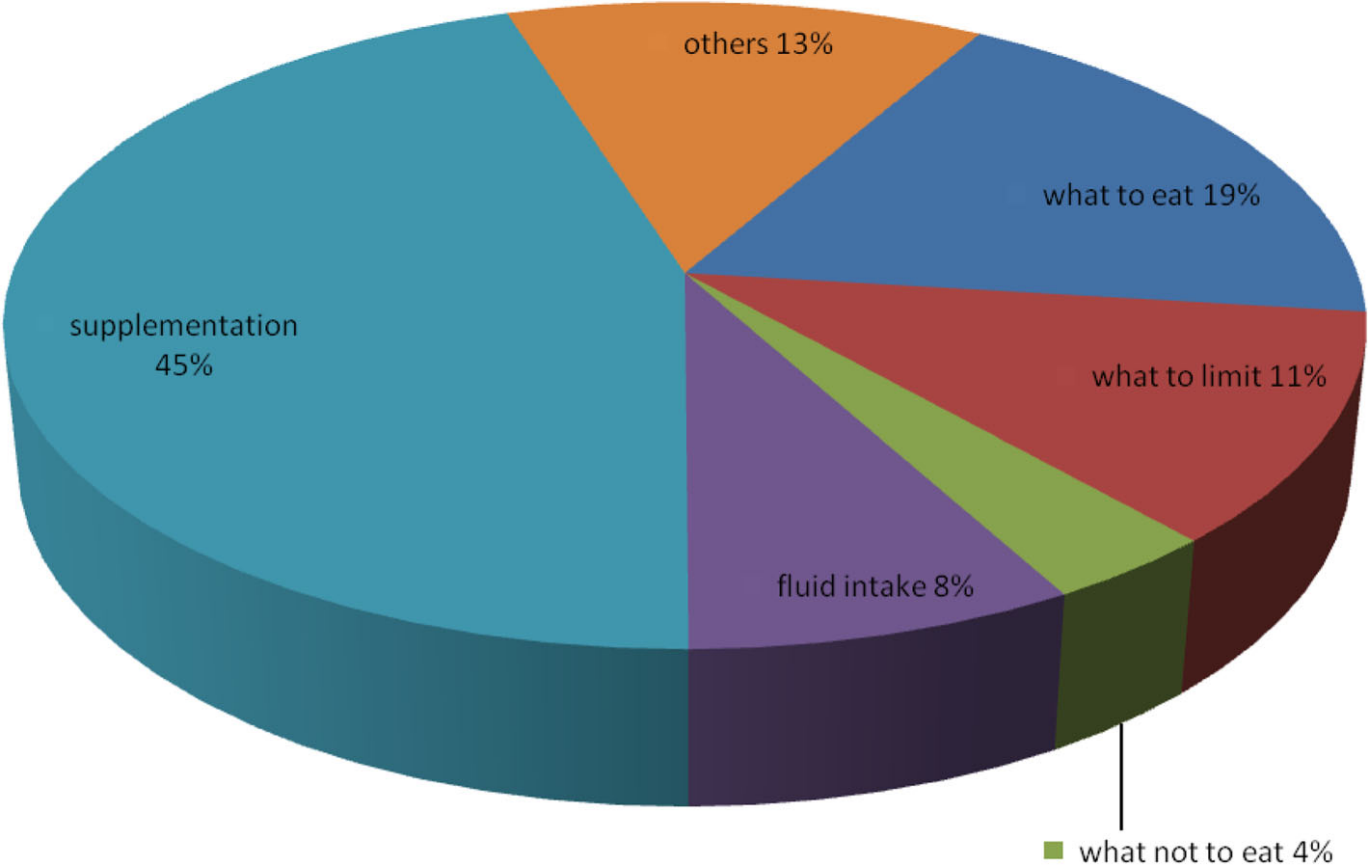
Nutrition related activities performed by health professionals

Most activities and counseling session’s relation to nutrition were related to supplements. Very few clients were informed about what not to eat during pregnancy. The importance of taking fluid during pregnancy was neglected even though it is very essential for both the mother and fetus, Sufficient information had not been provided to mothers about what to limit to eat during their pregnancy (Fig. 1).

**Fig. 2:**
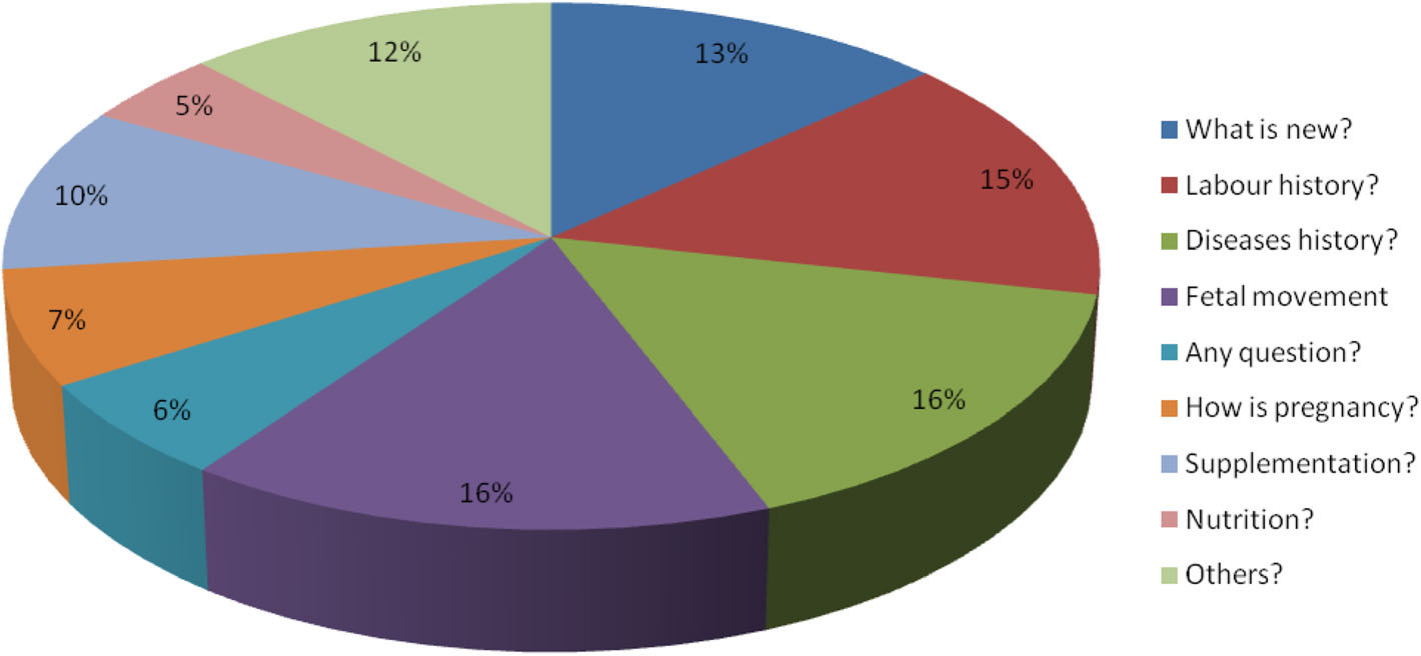
main questions rose by health professionals to mothers

**Fig. 3:**
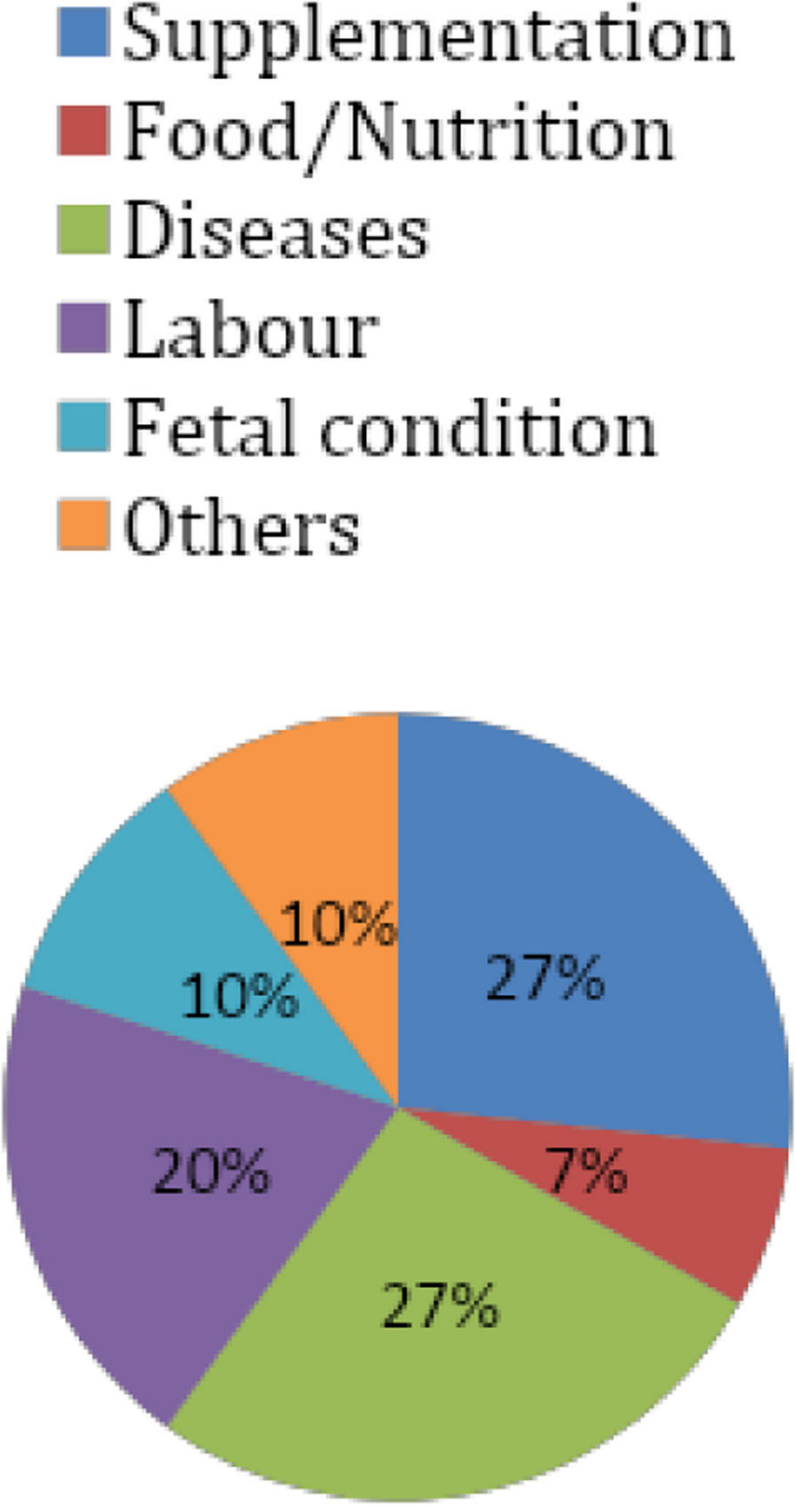
Major questions rose by pregnant mothers to health professionals

It is obvious that professionals’ concern is, very few in relation to nutrition. Their main concern is about fetal condition, the health status of the mothers, and the labor history of mothers. They would like to ask the mother gross questions like “*what is new*?” rather than specific questions about diet and food. On the other hand, we can observe that mothers’ information-seeking behavior is very poor. Only a few questions were raised by pregnant mothers related to nutrition and food. Most questions were relating to their health status and disease condition in addition to their fetal condition. Mothers pay more attention to supplements than food and nutrition. So, nutrition is a neglected subject by the mothers as well (Figs. 2 and 3).

From the environmental observation, the majority of the communication channels are wall-mounted displays both in the waiting areas and ANC OPD examination rooms. On the other hand, the majority of the information displayed and transmitted was related to disease conditions and treatment protocols. Many of the messages posted and displayed are less visible from distance. Surprisingly, no information was displayed, and posted in relation to maternal nutrition and diet system. On the other hand, the observer has experienced no nutrition mass-education session during the whole observation period (Tables 4 and 5).

**Table 4:** Environmental observation of waiting for areas

Channel of Communication	Types of Message displayed or transferred	Visibility from distant (> 2.5 m)	Color and size Category
Notice-board	Different notices concerning staffing and clients	Less	White papers and small
Self-stand Chart	Danger signs during pregnancy in Amharic	Visible	Colored and large
Self-stand Chart	Procedures with price list	Visible	Colored and large
Self-stand Chart	Feedback and free call line	Moderate	Colored and medium
Self-stand Chart	Service lists	Visible	Colored and large
Self-stand Chart	Service lists	Visible	Colored and large
Television	Family planning methods, Stage of growth and fetal movement	Visible	Colored and medium
Wall mounted Chart	Case definition of Ebola in Amharic	Less	White papers and small
Wall mounted Chart	Danger signs during pregnancy in English	Less	White papers and small
Wall mounted Chart	Danger signs during labor in English	Less	White papers and small
Wall mounted Chart	Suspected case definition of AWD	Moderate	White papers and small
Wall mounted Chart	Case definition of AFP	Moderate	White papers and small
Wall mounted Chart	Case definition of Meningitis	Moderate	White papers and small
Wall mounted Chart	Case definition of neonatal tetanus	Moderate	White papers and small
Wall mounted Chart	Stage of fetal growth	Visible	Colored and medium
Wall mounted Chart	Case definition of Ebola in Amharic	Less	White papers and small

**Table 5:** Environmental observation of ANC OPD examination rooms

Channel of Communication	Types of Message displayed or transferred	Visibility from distant (> 2.5 m)	Color and size Category
Book Shelf	Different topics	Visible	Colored and white
Notice-board	Different notices concerning staffing and clients	Less	White papers and small
Wall mounted Chart	Management of Convulsion	Moderate	Colored and medium
Wall mounted Chart	Family planning methods	Visible	Colored and medium
Wall mounted Chart	Stage of fetal growth	Visible	Colored and medium
Wall mounted Chart	Hand washing procedure	Less	White papers and small
Wall mounted Chart	Traditional ANC periods	Less	White papers and small
Wall mounted Chart	Focus ANC periods	Moderate	Colored and medium
Wall mounted Chart	Laboratory result references	Less visible	White papers and small
Wall mounted Chart	Mgso4 injection protocol	Less	White papers and small
Wall mounted Chart	Syndrome diagnosis if STI	Moderate	Colored and medium
Wall mounted Chart	2016 WHO ANC Model	Less	White papers and small
Wall mounted Chart	Clinical stage of HIV/AIDS	Moderate	Colored and medium
Wall mounted Chart	Emergency maternal condition management	Moderate	Colored and medium
Wall mounted Chart	Stage of fetal growth	Moderate	Colored and medium

### Interview

The interview participants include term pregnant mothers with a mean age of 31 years; the number of pregnancies 2.4 and gestational age 37.8. Most health professionals were males. The two center managers have an average experience of 7 years.

The result of the study is summarized into the following main categories: Adequacy of maternal nutrition, when to provide, where to provide, by whom to be provided, types of information, other sources of information, barriers, and possible solutions of maternal nutrition counseling (Table 6).

**Table 6:** Profiles of interview participants

Pregnant Mothers			
Participants	Gestational age	No. of Pregnancy	Educational level
1	37	4	Tertiary
2	38	4	Tertiary
3	37	3	Tertiary
4	37	3	Tertiary
5	38	3	Secondary
6	38	2	Tertiary
7	38	2	Secondary
8	39	2	Primary
9	39	1	Tertiary
10	38	1	Tertiary
11	37	1	Secondary
Average	37.8	2.4	
Health professionals			
Participants	Qualification	Total Experiences	
1	Senior Gynecologist	22	
2	Gynecologist	9	
3	Senior Gynecologist	15	
4	Emergency Surgical Officer	9	
5	Emergency Surgical Officer	15	
6	Medical Doctor (GP)	2	
7	Medical Doctor (GP)	2	
8	Senior Gynecologist	25	
9	Senior Gynecologist (CM)	27	
Average		14	
Center Managers			
Participants	Qualification	Total Experience	
1	Master of Public Health	8	
1	Medical Doctor	6	
Average		7	

### Adequacy of maternal nutrition counseling

Most participants respond that maternal nutrition counseling provided to pregnant mothers is inadequate. Among mothers, eight of them replied that maternal counseling provided to them is not adequate; one of them stated that; it is adequate, whereas three of them responded that; maternal nutrition counseling provided by health professionals is fair enough. All health professionals and health center managers responded in the same context and strongly and loudly said; it is difficult to say that maternal nutrition education and counseling provided by health professionals is adequate. They responded that whatever indicator we use, maternal nutrition education and counseling provided during pregnancy couldn’t be stated as adequate. This again has three subcategories:

Time: No or very short time, Content: Most of the study participants responded that; most often gross, less detail and very little maternal nutrition and Mothers’ understanding dimension: Most health professionals responded that since there is very short time allocated to each mother, it is difficult to counsel mothers up to their advanced level of understanding.

*“I think the information about nutrition given to us during our ANC visit is fair enough. They told us about our feeding practice. For example, they told us the important food items for the fetus’s growth. They told me how often to eat per day. They told me that the frequency of feeding should be more than the regular time of eating practice”* (Interview with pregnant mothers, M3).

*“Firstly, I have never given education and counseling about maternal nutrition. I directly came to the institution and told them that I want to have an ANC follow-up and started immediately. But during the whole period of my ANC follow-up, no one gave me the information about nutrition”* (Interview with the pregnant mother, M5).

*“No information is provided to me about maternal nutrition counseling in my ANC visit from doctors. I didn’t remember anything related to nutrition counseling and education during the whole ANC follow-up”* (Interview with pregnant mothers, M7).

*“I feel that maternal nutrition education and counseling provided to pregnant mothers is not adequate. Why it is not adequate; one if we see in the respective of time, especially in our setup, for example, we have a lot of clients each day. So it is difficult to tell mothers what to eat, how much to eat, how often to eat per day and other nutrition-related issues”* (Interview with senior Gynecologist, P2).

*“I don’t think so; it is not adequate. For example, if we measure, according to the time given to nutrition counseling sessions during pregnancy, it is not adequate. To counsel one pregnant mother about everything related to pregnancy, it requires thirty-forty minutes on average. But, in our setup, we counsel each mother for ten-fifty minutes. In our health institution, mothers coming for ANC follow-up have different educational statuses. There are mothers whose educational levels are primary, secondary, and tertiary. Mothers of tertiary educational level have a high understanding capacity for any counseling provided to them. Those of the only primary educational level may have a lower understanding capacity during counseling. Normally, it is important to counsel mothers on what content of food to take like the quantity of carbohydrate, fat, minerals, proteins, vitamins, and water. But, we roughly tell mothers to take vitamins; fluids, protein, and others which only contain gross information. But, it is important to tell the details of these things to mothers. In general, maternal nutrition counseling and education provided to pregnant mothers are not adequate”* (Interview with the emergency surgical officer, P6)*.*

*“In my opinion, maternal education and counseling service provided by health professionals to pregnant mothers are not adequate. When are we going to measure the adequacy of this service using indicators like time, content, understanding of mothers, and others; for example, if we see regarding time given to nutrition counseling, there are more than 150, clients each day in our health facility. On the other hand, we have only four ANC OPD examination rooms. Having such a huge number of clients and very few health professionals, it is so difficult to offer proper nutrition counseling and education to each mother” (*Interview with center manager, CM2*).*

### When to provide it?

Among the eleven mother respondents, six of them said that; it is better to start at the first ANC visit and continue the process of counseling up to the end of pregnancy. Whereas, five of them replied that; it is possible to start at any time and exercise in each ANC visit. Among the health professional respondents, two of them believed that; it is better to start maternal education, and counseling at the preconception period and continue throughout the whole pregnancy period. Seven of these respondents agreed to start counseling and education in the first ANC visit and continue until mothers give birth. Two of the center managers stated that; the service should start at the first ANC visit and shall continue up to the end of pregnancy. Some participants are thinking about having a fixed day of maternal nutrition counseling among the ANC visits. Most others believed in providing mass-education to all mothers collected around waiting for areas in one of the days in a week.

*“Okay, nutritional counseling should be started at the time of the first ANC visit. It should be given by the gynecologist in the examination rooms. But, nutritional counseling should be given up to the end of the pregnancy in each ANC visit” (*Interview with the pregnant mother, M2*).*

*“This maternal nutrition counseling should be started before conception. Mothers should ask themselves that am I good enough to become pregnant? So, she has to have a complete medical diagnosis and examination. At that time, anemia, malnutrition, and other disorders could be checked” (*Interview with a senior gynecologist, P1*).*

*“For the question, when to provide maternal nutrition counseling, it must be offered at any time of mothers’ ANC visit. It is important to deliver this information at any visit of any pregnant woman. At least it is mandatory to give maternal nutrition counseling in the first ANC visit of every pregnant woman. Detail information should be provided to them in the first ANC visit”* (Interview with the gynecologist, P3).

### Where to provide it?

All the eleven participants responded that; maternal nutrition counseling and education should be provided in ANC OPD examination rooms by the same health professionals who provide other ANC service packages. Four of them underline that; it is best if given in a separate counseling room by the nutritionists. Three of them believe in providing it in an ultrasound room, triage, and waiting areas of the health institution. Among the participants of health professionals, seven of them strongly believed that; it is better to be provided in separate nutrition counseling rooms. Additionally, two of the health professionals agreed to provide it in waiting areas as well as together with other counseling sessions like family planning, HIV/AIDS, and others. Two of the center managers believed to provide the service in a separate room by the nutritionists. One of the center managers strongly underlined that; this service should be provided in every corner by all concerned health professionals to consistently bringing a behavioral change to mothers. On the other hand, some participants believe that; it is possible to provide it is everywhere like offices, market places, churches, and villages.

*“Counseling and education of maternal nutrition shall be given by the same doctor in the OPD examination rooms. After investigation of our laboratory result, doctors told our blood type, the fetal condition, ultrasound result, and food items and drinks to be taken and not. If nutrition professionals assigned in a separate room to offer nutrition counseling to mothers, it is the best and most appropriate way of information delivery to mothers”* (Interview with the pregnant mother, M6).

*We can use a projector and display nutritional issues around waiting areas of health institutions. Project display can assist mothers to discuss each other in waiting areas* (Interview with a senior gynecologist, P4).

*“It should be provided by all health care professionals at every corner in a consistent manner. Information provided to mothers should be consistent at all corners”* (Interview with center manager, CM1).

### By whom it should be provided?

Among the eleven participants of pregnant mothers, four of them believed the possibility of providing maternal nutrition counseling by the gynecologists as the full package of ANC services; the other four participants stated the provision of counseling and education by all health professionals working in ANC OPD examination rooms. Four pregnant mothers strongly agreed on the betterment of counseling if provided by nutritionists in separate counseling units. Three mothers stated that; health institutions could deliver the nutritional information in waiting for areas through different means with the assistance of health professionals or without the presence of them. Seven of the health professionals strongly agreed to the provision of maternal nutrition counseling by the nutritionists in separate counseling units. Two of them indicated the possibility of offering this service by the gynecologists in the same ANC OPD examination rooms. On the other hand, two of the health professionals underline the importance of mass-education in waiting areas by any health professional having nutrition training. Two of the center managers believed that; this maternal nutrition counseling should be provided by nutritionists in separate counseling units. (Table 7).

**Table 7:** When, where, and by whom nutrition counseling should be provided?

When?	Where?	By whom?	Mode of delivery
Immediately after conception In the first ANC visit Every ANC visit Preconception period	ANC OPD Waiting areas Separate counseling unit Ultrasound room Triage Every corner	Nutritionist Gynecologist All health professionals	Full ANC package Special ANC service separately Together with other counseling units

*“It is better to assign specific nutrition professionals in separate rooms. If nutrition profession is assigned, mothers can access relevant and sufficient information about maternal nutrition during pregnancy”* (Interview with a pregnant mother, M8).

*‘It is not the gynecologist, the triage, or other health professionals, the nutrition professional herself/himself should provide the appropriate nutritional counseling to mothers. For instance, let me take myself as an example, I don’t have sufficient knowledge as much as nutritionists have about it”* (Interview with a senior gynecologist, P2).

*“This education and counseling could be better given by health professionals who are educated and trained in nutrition. It is a fact that nutrition counseling and education could be provided by nutrition professionals better than other health professionals. But, it is possible to provide it by other health professionals like gynecologists, nurses, midwives, and others” (*Interview with center manager, CM2*).*

### Types of nutrition information to be provided

Almost all the respondents agreed upon the importance of nutrition counseling to both the mother and the fetus. Basic nutrition information should be provided to all pregnant mothers with special attention for the first pregnancy of the first ANC visit.

*“I believed that physicians should counsel the mothers how often, and how much to eat. Particularly, how to prevent nausea and vomiting in the first trimester of pregnancy should be informed to mothers by health professionals” (*Interview with the pregnant mother, M4*).*

*“At Minimum, we professionals should counsel mothers what to eat, what not to eat, how often to eat, and how much to eat during their ANC visit. This type of counseling does not consider pregnant women with special conditions like who have diabetes, obesity, and other nutrition-related cases” (*Interview with a senior gynecologist, P3*).*

*“Counseling should be based on the stage of pregnancy. In the first trimester, nutrition counseling should focus on the prevention and alleviation of certain disorders like nausea, vomiting, constipation, and heartburn which are related to pregnancy. Proper nutrition counseling based on maternal body mass index and fetal weight gains is vital in the reduction of pregnancy-induced maternal and fetal complications. In the second and third trimester, nutrition counseling and education in relation to varying and balanced for the maintenance of maternal health as well as for the growth and development of the fetus” (*Interview with a senior gynecologist, M7*)*.

### Other sources of nutrition information

This is to mean that source of information to pregnant mothers other than health professionals in health facilities. These could be divided into three basic categories. This again has four subcategories:

Community-based: Information from community members, elderly people, experienced friends, family members, neighbors, partners, peers, traditional birth attendants, and traditional healers are under the category of community-based sources of information.

Institution-based: Books, brochures, free-call centers, pamphlets, posters, projector display, messages through personal cell phone, and TV display in waiting areas are major institutional-based sources of nutrition information other than health professionals.

Media-based: Internet, magazine, mass-media, newspapers, and radio and TV programs are basic information sources for pregnant mothers in addition to health care professionals.

*“We can get nutritional information from the internet, from mothers who have previous experience, from reading books and from mass-media. In myself, I have got information from the internet and experienced mothers about maternal nutrition”* (Interview with the pregnant mother, M5).

*“As I said before, pregnant mothers can get nutrition-related information from their friends, from older people, from their mothers, from traditional healers, from traditional birth attendants, and other community members. Now the time is more globalized, technology-based and there is the ease of internet access. So, mothers can search for information from the internet as well as they can read books”* (Interview with a senior gynecologist, P1).

*“The other most important way of maternal nutrition information delivery method for me is by using free-call services. We as an organization have a free-call service providing information for clients about family planning, HIV/AIDS, danger signs, and others. These free-call service providers are actually nurses; so, we can train them about nutrition in detail, and they can provide nutrition information to any client calls to free-call center” (*Interview with center manager, CM2*).*

### Barriers to maternal nutrition counseling

Almost all the respondents agreed and emphasized that maternal nutrition counseling is totally neglected and forgotten by all stakeholders. According to the respondent’s perspective, there are many interwoven challenges hindering the provision of maternal nutrition counseling by health professionals to pregnant mothers. These barriers can be categorized into four sub-categories.

Institutional Barriers: these are the main challenges from the health institutions’ side.

Professional Barriers: Attitudes of professionals towards mothers’ understanding, the concentration of clinicians on danger signs, and management of other complications neglecting maternal nutrition, poor communication skill of health professionals, lack of knowledge and skill of health professionals as well as negligence and low attention of health professionals.

*“I believe that I am not good enough to provide proper maternal nutrition counseling and education to pregnant mothers during their ANC visit” (*Interview with a senior gynecologist).

Maternal Barriers: though pregnant mothers need information about nutrition in detail, there are few weaknesses from their side.

Community Barriers: Religion and culture, as well as the provision of non-scientific and harmful nutrition information to pregnant mothers, were the two main barriers according to the respondents who took part in the study. *“In my opinion, physicians neglect nutrition counseling issues. They may think that maternal nutrition has no strong impact on the process of pregnancy. On the other hand, a high number of clients and a shortage of time may be the main barrier to maternal nutrition counseling. Since physicians have many clients per day, they ignore nutrition counseling issues”.* (Interview with the pregnant mother, M1).

*“There are many barriers to provide maternal nutrition counseling. For example, from the health care provider’s side, the communication barrier is one of the main factors that hinder the provision of nutrition counseling. From the health facilities side, shortage of time because of high client flow is one main barrier. The other barrier of health institutions is there is no counseling guideline. From the clients’ side, as I said before, their educational status is one of the major barriers. Fewer acceptances of mothers to the counseling given to them by health professionals may be another barrier. Sometimes, clients may not listen properly to the words of doctors and vice versa. The economic status of mothers is another most important factor. After we deliver nutrition counseling to mothers, they may suffer due to lack of money to purchase the desired food items and supplements prescribed” (*Interview with the emergency surgical officer, P6*).*

*“I think the first one is the lack of attention to the subject. Food and nutrition issues are not that much supported by curriculum and training. The effect of this subject is inter-generational. A mother who is malnourished has a high probability of giving malnourished babies. The problem of nutrition recycles itself. So, the impact of nutrition could be on individuals, households, community, and country level. Though its impact is multi-faceted, it does not give due attention to our curriculum and training programs. On the other hand, we think about pregnancy and childbirth; we always consider danger signs and other complications rather than nutritional issues. In general, nutrition as a subject has no priority given by all stakeholders” (*Interview with center manager, CM1*).*

### Possible solutions for barriers to maternal nutrition counseling

The possible solutions have been categorized into five subcategories. Institutional, professional, maternal, community, and national levels of solutions are analyzed. Each possible solution should be established and exercised by all stakeholders to bring dramatic changes in maternal nutrition counseling and education process (Table 8).

**Table 8:** Possible solutions for maternal nutrition counseling

S. NO.	Solutions	Activities to be done
1	Institutional	Avail food composition table Avail nutrition counseling guideline Balance the number of professionals and clients/patients Establish separate nutrition counseling unit Combine with counseling units Distribute nutrition messages through personal phones Effective utilization of Media Enhance physicians motivation through trainings Establish free-call centers Include the course in all levels, including specializations Make the agenda priority of the ANC service package Provision of mass-education consistently Recruit and assign nutrition focal person Revised and update curriculum Set minimum-maximum client and time for each client
2	Professional	Exercise efficient time management Update timely nutrition information Provide client-specific nutrition counseling Involve partners in the counseling session Avoid communication barriers Tell the mothers about the right sources of information
3	Maternal	Educate and empower women Enhance information seeking behavior Refer and recheck the other sources of information Enhance self-pre-conception health assessment
4	Community	Create community awareness about maternal nutrition Educate and empower communities Discuss with community and religious leaders
5	Nationwide	Enhance gender equality and empower women Enhance the nations’ development and prosperity

*“First, the issue of maternal nutrition counseling and education should be realized and accepted as very essential to mother and then a separate counseling room with appropriate professional should be opened and assigned. It is very crucial if one specific nutrition profession is assigned in separate rooms for maternal nutrition counseling and education. The other most important thing is sending a text message to pregnant mothers through their personal cell phone” (*Interview with the pregnant mother, M6*).*

*“As an intervention; when mothers come to ANC visit, they should get appropriate nutrition counseling. Particularly, in their first visit, mothers should get nutrition counseling in a separate nutrition counseling unit. If it is not possible to recruit nutrition professionals, other professionals providing ANC services like gynecologists, midwives, general practitioners, and others should be trained very well to have sufficient nutrition and counseling skills, If possible institutions should give nutrition training periodically. The other thing pressure from religion should be discussed with religious leaders and solved. Another most important is the empowerment of mothers by education, and the economy. To access the recommended diet, during pregnancy, it is vital to have the potential to produce or to purchase food items. “When the economic status of the mother is poor, it is difficult and shameful to counsel about proper nutrition.” “As a gynecologist was not trained and built to have good knowledge of nutrition science and appropriate nutrition counseling skills” (*Interview with a senior gynecologist, P2*).*

*“At least as a solution, professionals should get ready to deliver the minimum maternal nutrition counseling within the short allocated period. On the other hand, brochure preparation, and distribution might be one key solution to deliver maternal nutrition information. If possible, a separate nutrition counseling room by nutrition professionals is the best option. But, I think it is difficult to do so. On the job training to professionals who deliver ANC service could be another important solution”* (Interview with a gynecologist, P3).

*“Professionals should update themselves timely and accordingly better than clients. On the other hand, the number of clients assigned to each professional should be limited. If the numbers of clients assigned to each health professional become limited, professionals can have time to counsel important issues during pregnancy including maternal nutrition. I didn’t remember a nutrition session in postgraduate (specialization) programs. This course was given to us in an undergraduate class in the title “pediatric health and nutrition.” Its emphasis was more on infants, and children’s nutrition, not maternal nutrition. But, it is in general one aspect of the ANC package. So, it is better to have lectured in post-graduate classes (specialization programs). Or on job training could be given to professionals working in the ANC OPD. If on-the-job training is given. It is best for professionals to deliver appropriate and timely nutrition counseling to mothers. Sometimes, mothers may have sufficient availability and accessibility of food; but due to lack of appropriate information about nutrition and diet, they may miss their chance of proper nutrition and appropriate diet systems. Nutrition counseling could be given to each mother, according to their economic status”* (Interview with a senior gynecologist, P4).

**Fig. 4:**
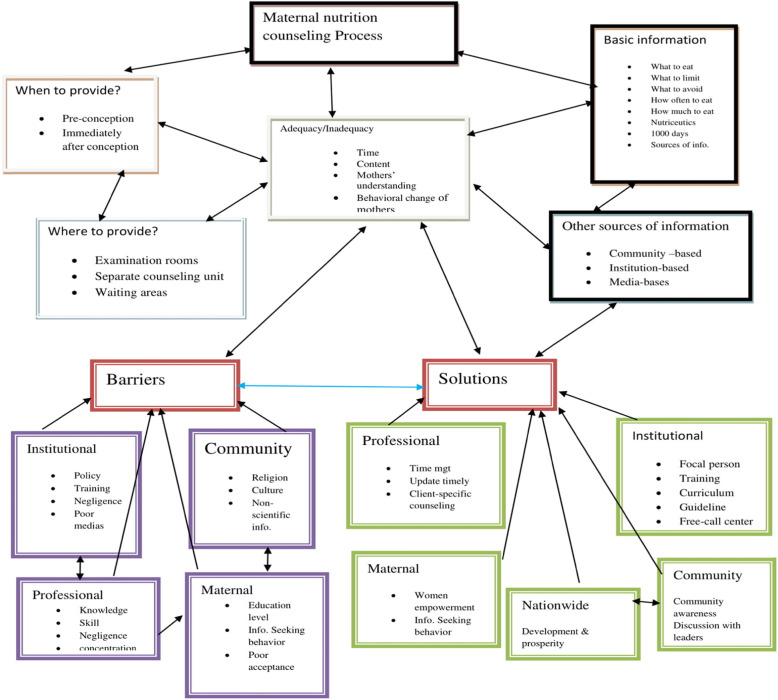
Conceptual framework for maternal nutrition counseling drown from this research

A Conceptual framework for maternal nutrition counseling drowns from this research (Fig. 4).

## Discussion

The provision of counseling about a balanced diet and its importance during pregnancy is mandatory. The discussion about locally held beliefs, attitudes, and misperceptions on foods considered healthy or appropriate for consumption during pregnancy and provide recommendations on culturally appropriate healthy eating interventions, based on the local context are integral to the health of the mother and the wellbeing of the children [16], [4].

From practical site observations, maternal nutrition counseling was very low. This might indicate that; maternal nutrition counseling has got less attention from all concerned bodies. Nothing is done or said how to relieve heartburn and constipation. Only one mother had been counseled how to relieve nausea during the whole observation period of pregnant mothers’ ANC visit highly indicating the inadequacy of maternal nutrition counseling during pregnancy, which similarities to few studies and article reviews [13], [15], [17], [18], these might be due to most health professionals, health institutions, or even the government gives less attention for maternal nutrition counseling.

The result of this study revealed that maternal nutrition education and counseling provided during ANC follow-up is not adequate in general. From the observation part, it is obvious; the issue of maternal nutrition is neglected by all stakeholders. Health facilities, professionals as well as mothers were mainly concerned about maternal health status, and fetal condition. Only very few questions were raised by both professionals and pregnant mothers about food and nutrition in all observation parts of the investigation. Messages posted and displayed were out of the issue of maternal nutrition; besides their invisibility from distance. Even though it was difficult to get health-related research done through practical site observation of pure qualitative study, the investigator got more emphasis on the negligence of the subject by observing the situation under which maternal nutrition is expected to be conducted which is similar to a study conducted in Ethiopia [19].

In this study, though we use many triangulation methods, evidence collected couldn’t reveal the adequacy of maternal nutrition education, and counseling provided by health professionals to pregnant mothers. Using parameters of time, the content of information, and understanding of mothers; the subject is not adequately addressed which indicates similarity with studies conducted in Senegal in 2019 and systematic review done in south-east Asians in 2017 [15], [20].

According to the review of articles done in June 2017 in UAS, despite the opportunity to counsel mothers on diet during pregnancy at routine health facility visits, there is limited available evidence on the type and quality of information and counseling received on maternal nutrition and weight gain during pregnancy that is very similar to the findings of this study in which no documentation is found about maternal nutrition counseling [2].

Some physicians believed that it is better if given during the preconception period. But, in the absence of preconception assessment programs, it is better to start counseling immediately after conception in the first ANC visit of mothers and continue up to the whole period of pregnancy till mothers give birth.

Most respondents stated deeply about the betterment of providing maternal nutrition counseling by nutrition professionals in a separate counseling room individually to each mother as well as underline the issue of providing mass nutrition education in waiting areas of health facilities. According to many of the study participants; it is very fantastic if given by trained nutritionists in separate counseling rooms. But, it is OK to provide nutrition counseling by health professionals working in ANC OPD examination rooms and all health professionals at every corner provided the quality, quantity, and consistency of the information offered.

The result of this study indicated the most important maternal nutrition information should be concerned about what to eat, what not to eat, what to limit to eat, how much to eat, how often to eat, nutriceutics, the first 1000 days, and the right sources of information for mothers.

Experienced friends, family members, neighbors, partners, peers, traditional birth attendants, and traditional healers from the community members, Books, brochures, free-call centers, pamphlets, posters, projector display, messages through personal cell-phone and TV display in waiting areas from institutions, the internet, magazine, mass-medias, newspapers, and radio and TV programs from Medias are considered the main sources of information for pregnant mothers other than health professionals.

Research conducted in Australia in 2017, stated that, although midwives perceived providing nutrition advice to pregnant women as an integral part of their practice as midwives, this role was felt to be constrained by many challenges and factors mostly out of the midwives’ control which is similar with the result of this study [5].

From the in-depth interview part of this study, most studying participants respond that maternal nutrition education and counseling provided to pregnant mothers are not adequate. Whatever the indicator we use, its adequacy is under a question mark. The study participants clearly stated the main challenges of maternal nutrition counseling. According to most participants, shortage of time, negligence of health professionals, lack of counseling guideline materials, lack of training, curriculum problems, and mothers’ level of understanding is the main constraint for nutrition counseling which similar to the research conducted in Uganda in 2018 [14].

According to the study done in Ethiopia, although there is a significant relationship between nutrition information and nutrition practices of mothers during pregnancy, the pregnant mothers’ access to nutritional information is relatively low which is very similar to the inadequacy of maternal nutrition counseling of the study [18].

Balancing the number of clients and health professionals, availability of nutrition counseling guidelines, timely training, providing client-specific counseling, the establishment of free-call centers, message through a brochure, pamphlets, personal cellphone, empowerment of women in particular and the community in general, it is stated as the main possible solutions from multiple alternatives for the barriers of maternal nutrition counseling.

The current nutrition counseling process is inadequate when measured regards time, the content of information, and the understanding level of mothers. The study clearly showed when to provide, where to deliver and by whom it could be better provided which indicates the time, place, and professional dimension for nutrition education, and counseling provision. Additional, important nutritional information which is not forgettable to all pregnant mothers is clearly found out. Possible sources of information other than health professionals to pregnant assessed in detail. Major barriers to nutrition counseling, which is the focus of many types of researches, are explored in detail from different perspectives. Not only major barriers but also possible interventions are explained clearly with their stakeholders.

Finally, the main essence of this study is to establish a conceptual framework based on the data collected. This is a substantive-micro-level a conceptual framework of grounded theory. If further quantitative research and analysis of the results are done, it can be reached to a normative- macro-level conceptual framework of grounded theory.

## Conclusions

This study concludes that; maternal education and counseling are not adequate, it lacks concentration, or didn’t conduct as a whole. Which means it provided either in the same examination room as a full package of ANC services by any health professional which indicates nutritional counseling to mothers by health professionals lack focusing by health institution managers, other health professionals, and stakeholders. As atone of study participants nutrition education and counseling for pregnant mothers organized if it is provided on examination rooms and waiting areas of health facilities What to eat, what not to eat, what to limit to eat, how often to eat, how much to eat and nutriceutics are among the most important kinds of information that should be provided to all pregnant mothers.

Community members, media, and electronic devices could be used to transfer nutritional messages to mothers. Shortage of time, absence of guidelines, lack of training, mothers’ level of understanding, negligence of all stakeholders, economic status of mothers, lack of knowledge and skill of health professionals, inefficient media, absence of policy, and poor curriculum are major barriers to nutrition counseling. Balancing clients and health professionals, timely training, women empowerment, making the agenda priority of stakeholders, effective utilization of media, curriculum revision, development, and prosperity of nations are some of the interventions among the many alternatives.

### Strength and limitation of the research

Most researchers reviewed are concerned about the barriers to nutrition education and counseling. But, this study is quite different in such a way that; it tried to address the process of a full package of maternal nutrition education and counseling in different health sectors. It touched from the current status of the counseling process up to possible interventions. This study tried to incorporate the primary beneficiary and stakeholders. The investigator tried to dig out the root causes and list all possible multi-dimensional interventions.

Practical observation content was done with a sense of making hidden agenda of the main subject matter; that is, maternal nutrition was observed in the context of the full ANC service package to prevent participant bias which seems contradictory to research ethical issues.

### Recommendations

Researchers: Quantitative or mixed research should be conducted to quantify the representativeness of the result obtained from this a pure qualitative study.

Health professionals: Health professionals should focus on the importance of maternal nutrition counseling, consider it as one of the main ANC service packages, and update themselves timely from different sources.

Health institution managers: since institutions are directly or indirectly concerned with the health of the nation, the managers should prepare timely and continuous training for health professionals related to nutrition. Health facilities should display nutritional information in every corner using different channels.

Media: taking the high dissemination power of information through media into account, the media should be played their own role in building the nation through proper nutrition.

Ministries of Education: Curriculum should be revised to include health and nutritional counseling for pregnant mothers in educational sectors. More health professionals are still required to balance between the clients/patients and health care providers.

To regional and federal governmental bodies: since the socioeconomic status of pregnant mothers described as a barrier to nutrition counseling working to increase the economical and social status of the mothers and the community as a whole by discussing with community leaders to bring nationwide development and prosperity.

## Data Availability

The datasets used and/or analyzed during the current study are available from the corresponding author on reasonable request through the following address agumlt@yahoo.com // fentahun143@gmail.com.

## Abbreviations

ANC: Ante Natal Care
BMI: Body Mass Index
CBC: Complete blood count
EDHS: Ethiopian Demographic and Health Survey
FGAE: Family Guidance Association of Ethiopia
GWG: Gestational Weight Gain
MCH: Maternal and Child Health
NEC: Nutrition Education and counseling
NGO: Non-governmental Organization
NNP: National Nutritional Program
OPD: Outpatient Department
SDG: Sustainable Developmental Goal
WHO: World Health Organization
